# The Altered Anatomical Distribution of ACE2 in the Brain With Alzheimer’s Disease Pathology

**DOI:** 10.3389/fcell.2021.684874

**Published:** 2021-06-25

**Authors:** Huan Cui, Si Su, Yan Cao, Chao Ma, Wenying Qiu

**Affiliations:** ^1^Department of Human Anatomy, Histology, and Embryology, Neuroscience Center, School of Basic Medicine, Institute of Basic Medical Sciences, Chinese Academy of Medical Sciences, Peking Union Medical College, Beijing, China; ^2^Chinese Institute for Brain Research, Beijing, China

**Keywords:** ACE2, brain regions, Alzheimer’s disease, COVID-19, anatomical distribution

## Abstract

The whole world is suffering from the coronavirus disease 2019 (COVID-19) pandemic, induced by severe acute respiratory syndrome coronavirus 2 (SARS-CoV-2) through angiotensin-converting enzyme 2 (ACE2). Neurological manifestations in COVID-19 patients suggested the invasion of SARS-CoV-2 into the central nervous system. The present study mapped the expression level of ACE2 in 12 brain regions through immunohistochemistry and detected ACE2 in endothelial cells and non-vascular cells. The comparison among brain regions found that pons, visual cortex, and amygdala presented a relatively high level of ACE2. In addition, this study demonstrates that the protein level of ACE2 was downregulated in the basal nucleus, hippocampus and entorhinal cortex, middle frontal gyrus, visual cortex, and amygdala of the brain with Alzheimer’s disease (AD) pathology. Collectively, our results suggested that ACE2 was expressed discriminatorily at different human brain regions, which was downregulated in the brain with AD pathology. This may contribute to a comprehensive understanding of the neurological symptoms caused by SARS-CoV-2 and provide clues for further research on the relationship between COVID-19 and AD.

## Introduction

From December 2019, novel coronavirus disease 2019 (COVID-19) characterized by respiratory failure has become a global and historical disaster (Guan et al., 2020). The pathogen, severe acute respiratory syndrome coronavirus 2 (SARS-CoV-2), interacts with angiotensin-converting enzyme 2 (ACE2) to infect, and make dysfunctional of the target cell (Kuba et al., 2005; Hoffmann et al., 2020; Hu et al., 2021). ACE2, one necessary member of the renin–angiotensin system (RAS), was first sequenced and identified from human heart failure (HF) ventricle and human lymphoma cDNA libraries. The RAS is classically seen as an enzymatic cascade, in which angiotensinogen is cleaved by renin to release angiotensin I (Ang I). ACE cleaves Ang I to form angiotensin II (Ang II) (Donoghue et al., 2000). The catabolites’ biological function of Ang II includes vasoconstriction, vasopressin release, induction of transcription factors, salt appetite, and drinking response, mainly mediated by AT1 receptors (Veerasingham and Raizada, 2003). ACE2 regulates the RAS by counterbalancing ACE activity through resolving Ang II into angiotensin 1–7 [Ang (1–7)]. Recent studies demonstrated that reducing ACE2 expression might promote the lung’s inflammatory process and the subsequent cytokine storm in many severe COVID-19 patients (Rodrigues Prestes et al., 2017; Banu et al., 2020; Wang et al., 2020). After being infected by SARS-CoV-2, ACE2 was downregulated and lost its protective function, contributing to organ damage.

All ACE2-positive tissues, such as lung, testis, and kidney, might be invaded by SARS-CoV-2, which furtherly evoke relative symptoms (Hamming et al., 2004). Although COVID-19 is considered a respiratory disease primarily, clinical evidence suggested the central nervous system in COVID-19 patients was affected, such as headache, epilepsy, and disturbed consciousness. In fact, some neurologic symptoms, especially anosmia and dysgeusia, occurred even before characteristic COVID-19-related symptoms (Avula et al., 2020; Baig et al., 2020; Jarrahi et al., 2020; Khan and Gomes, 2020; Liguori et al., 2020; Mao et al., 2020; Wu et al., 2020). In addition, SARS-CoV-2 was found in the cortical neurons in brain autopsy from patients who died of COVID-19 with pathologic features associated with virus infection. SARS-CoV-2 was also detected in infected patients’ cerebrospinal fluid, supporting that SARS-CoV-2 might directly attack cells in the brain, especially the ACE2-positive population. Recent studies detected ACE2 in the rodent brain, mainly located in the neural cells, and simulated the process of SARS-CoV-2 infection in human brain organoids (Doobay et al., 2007; Song et al., 2021). However, the presence and specific role of ACE2 in the human brain is still unknown. Therefore, mapping the cerebral distribution of ACE2 provides the basis for controlling the neurological symptoms and alerts to the potential risk.

Among comorbidities of COVID-19 in the central nervous system, Alzheimer’s disease (AD) has become a rising concern (Fotuhi et al., 2020). AD is a neurodegenerative disorder that affects mainly the memory and learning of the patients. As AD management does not fit with the isolation and quarantine management of COVID-19, COVID-19 loads extra burden on AD patients. In addition, due to cognitive impairments and degenerated physiology, AD patients are vulnerable during this COVID-19 crisis (Mok et al., 2020; Rahman et al., 2021). In the brain with AD pathology, the broken blood–brain barrier and dysregulated immune environment reflect a higher risk of virus infection and tissue damages. However, it is still controversial whether the distribution of ACE2, the receptor of SARS-CoV-2, was altered among AD pathology (Kehoe et al., 2016; Lim et al., 2020). In this study, we systemically compared the ACE2 expression in 12 brain regions in the brains comprising AD-related pathology or not.

## Materials and Methods

### Sample Sources

The brain samples were recruited from the National Human Brain Bank for Development and Function, Chinese Academy of Medical Sciences, and Peking Union Medical College (PUMC) in Beijing, China. Each brain sample was disposed of based on the standardized protocol of the human brain bank in China. The “ABC” scoring system was applied for the AD-featured neuropathological rating of human brains (Hyman et al., 2012). Paraffinic sections, including superior temporal gyrus, anterior cingulate cortex, inferior parietal lobule, pons, midbrain, amygdala, visual cortex, middle frontal gyrus, hippocampus and entorhinal cortex, basal nucleus, medulla, and cerebellar cortex and dentate nucleus from five AD donors and five age-matched non-AD donors, were used to detect ACE2 expression. To exclude the disturbance of gender, only female donors were absorbed into this study. The age and post mortem interval were compared between control and AD group: age, 84 ± 0.7071 vs. 76 ± 9.322, *P* = 0.4170; post mortem interval, 7.8 ± 2.453 vs. 10.92 ± 3.334, *P* = 0.4726. Detailed information of all the donors is listed in Supplementary Table 1. Written informed consent was obtained either from the donor or a close relative. The research protocol was approved by the Institutional Review Board of the Institute of Basic Medical Sciences of the Chinese Academy of Medical Sciences, PUMC, Beijing, China (approval number: 009-2014).

### Neuropathological Classification

**FIGURE 1:**
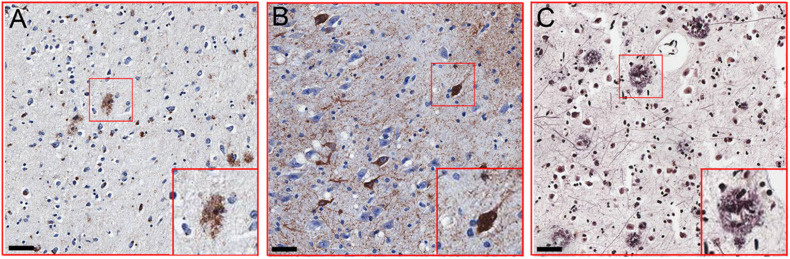
Representative images of Alzheimer’s disease (AD)-related pathology. **(A)** Immunohistochemical detection of β-amyloid plaques using 6F3D antibody in the hippocampus and entorhinal cortex (PTB297). **(B)** Immunohistochemical detection of neurofibrillary degeneration using a p-Tau antibody (Ser202 and Thr205) in the hippocampus and entorhinal cortex of (PTB 297). **(C)** Immunohistochemical detection of neuritic plaques in inferior parietal lobule (PTB297). Scale bar = 50 μm.

All participants received identical neuropathological evaluations by ADNC system according to 2012 National Institute on Aging-Alzheimer’s Association guidelines (Hyman et al., 2012). Briefly, all subjects were assigned the A score of Aβ deposits, the B score of neurofibrillary tangles, and the C score of neuritic plaques. Representative images for the staining of Aβ deposits, neurofibrillary tangles, and neuritic plaques are shown in Figures 1A–C. These three scores were summarized to determine the existence and degree of Alzheimer’s pathology. The detailed pathological information of each case was also listed in Supplementary Table 1.

### Immunohistochemistry and Immunofluorescence Staining

Serial sections were transferred into xylene for deparaffinage, and sequential 100, 95, 95, and 80% ethanol for rehydrating. Sections were treated with microwave heat-induced antigen retrieval (95°C, 30 min) and then were transferred into 5% hydrogen peroxide to block endogenous peroxidase. After blocking with 5% normal horse serum, sections were incubated with primary antibody (Rabbit anti-ACE2, 1:400, Abcam) at 4°C overnight. Then, sections were incubated with proper secondary antibodies (Goat Anti-Rabbit IgG H&L, 1:1000, Abcam). The negative control, the same volume of PBS buffer, was applied to test the antibody’s effectiveness. Images were acquired by laser confocal microscopic imaging system (FV1000 and Olympus FluoView software, Olympus, Japan). Ten representative views for each slice were selected to measure the mean optical density (MOD) by Image J, and the average of MOD was applied to evaluate the expression of ACE2. Endothelial cell was identified based on the circle-like appearance. The total numbers of ACE2^+^ non-vascular cell and non-vascular cell nucleus within each view were manually counted and summarized, and the percentages of ACE2^+^ cell in each brain region were calculated.

For immunofluorescence staining, serial sections were transferred into xylene for deparaffinage, and sequential 100, 95, 95, and 80% ethanol for rehydrating. Sections were treated with microwave heat-induced antigen retrieval (95°C, 30 min). Sudan black B (5%) was applied to remove autofluorescence of brain tissue and then was transferred into 5% donkey serum for 1 h. Primary antibodies (Rabbit anti-ACE2, 1:400, Abcam, ab15348; Mouse anti-NeuN, 1:500, Abcam, ab104224; Goat anti-GFAP, 1:500, Abcam, ab53554; and Goat anti-IBA1, 1:1000, Abcam, ab5076) were incubated at 4°C overnight in a wet box. After washing the sections by phosphate buffer saline (PBS), corresponding secondary antibodies were incubated for 1 h at room temperature. The stained sections were examined by laser confocal microscopic imaging system (FV1000 and Olympus FluoView software, Olympus, Japan). The CA1 and CA4 regions for each case were captured, and percentages of ACE2-immunopositive cells among marked cellular type (NeuN for neuron, GFAP for astrocyte, and IBA1 for microglia) were summarized.

### Statistical Analysis

Data analysis was performed by using the Prism 7.0 statistical program (GraphPad Software, Inc.). Shapiro–Wilk test was applied to determine the normality for parametric test. The difference between the Control group and the AD group was determined by Student’s *t*-test. The criterion for statistical significance was *P* < 0.05.

## Results

### The Distribution of ACE2 in Brain Regions

**FIGURE 2:**
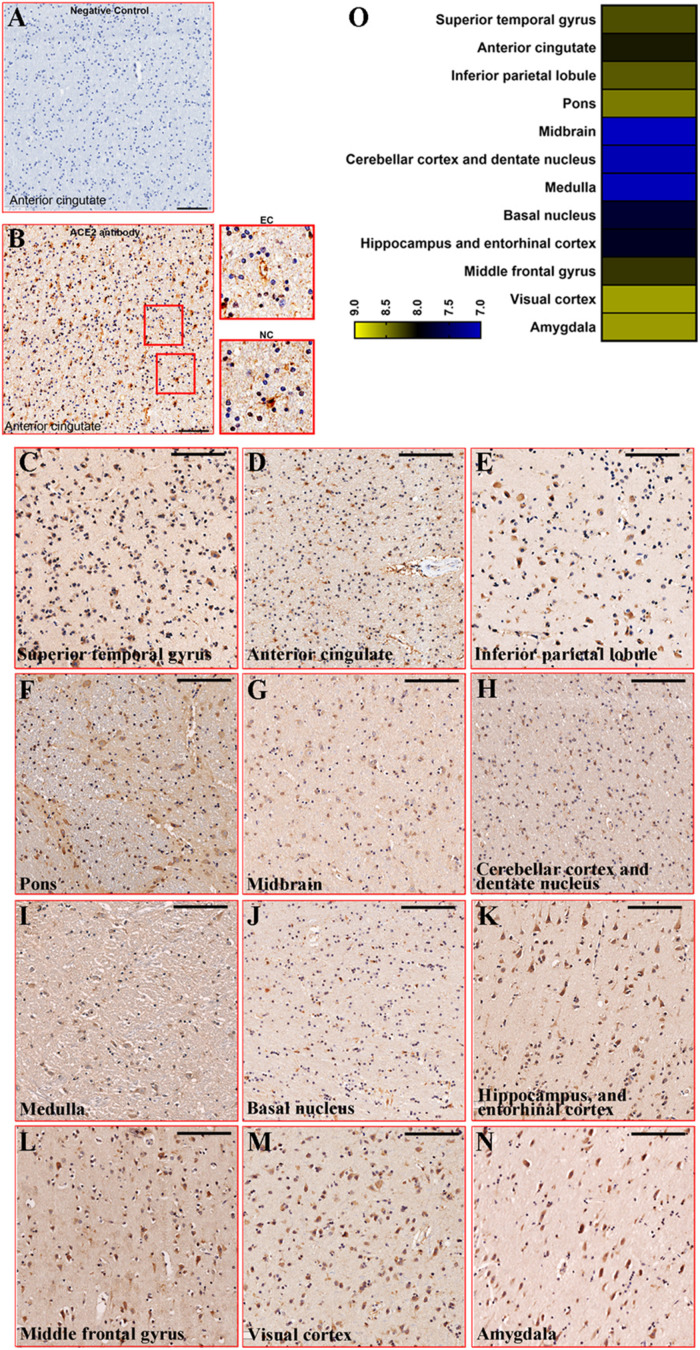
Representative images of ACE2 expression among brain regions. **(A)** Immunostaining of negative control for ACE2 in the anterior cingulate cortex (PTB270). Scale bar = 100 μm. **(B)** Immunostaining of ACE2 by Rabbit anti-ACE2 antibody in the anterior cingulate cortex (PTB270). Scale bar = 100 μm. The enlarged views were listed on the right. EC, endothelial cell; NC, non-vascular cell. **(C–N)** Representative images of ACE2 expression among brain regions in health control. Scale bar = 100 μm. **(O)** Heatmap showed the average of MOD in each brain region for the five control subjects. The legend scaled the average of MOD.

Firstly, we carried out the negative control of the ACE2 antibody in the anterior cingulate cortex to test its effectiveness. A clear positive signal was detected in the slice stained by the ACE2 antibody, while there was no signal in the slice stained by the buffer. In addition, the positive signal is mainly located in two cellular types, endothelial cell and non-vascular cell (Figures 2A,B). Based on the branching form, the non-vascular cell was mainly neuronal cells. The ACE2 expression was detected in all 12 brain regions of 5 healthy control subjects, while the intensity varied among brain regions. In detail, ACE2 was expressed at a high level in the pons, visual cortex, and amygdala, which was relatively reduced in the midbrain, cerebellar cortex, dentate nucleus, and medulla. The representative images of ACE2 expression among brain regions of healthy control subjects are listed in Figures 2C–N. The relative expression of ACE2 among brain regions was rated by heatmap in Figure 2O.

### Decreased Expression of ACE2 in the Brain With AD-Pathology

**FIGURE 3:**
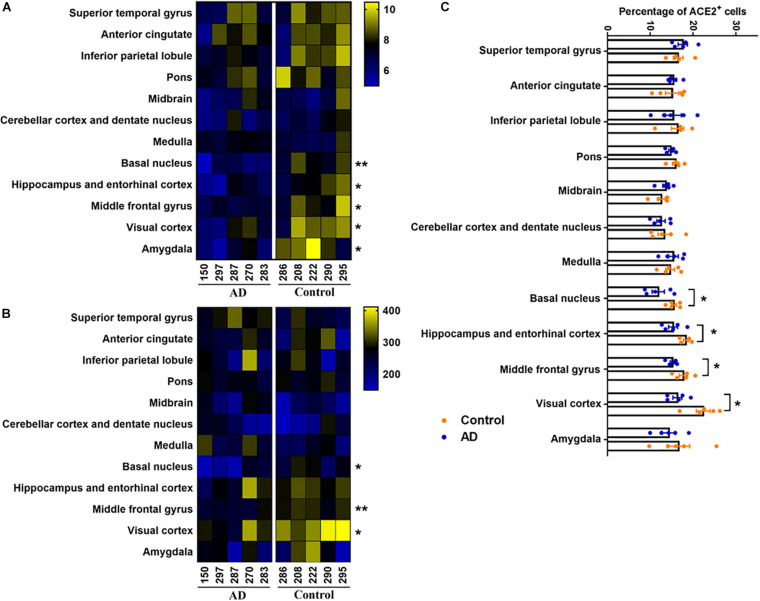
Comparison of ACE2 expression between Control and AD group by heatmap. **(A)** Heatmap showed the average of MOD in each brain region for the five AD subjects (left panel) and five control subjects (right panel), *n* = 5, Student’s *t*-test, *p < *0.05*, **p < *0.01*, AD vs. Control group. **(B)** Heatmap showed the number of ACE2 positive non-vascular cell in each brain region for the five AD subjects (left panel) and five control subjects (right panel), *n* = 5, Student’s *t*-test, *p < *0.05*, **p < *0.01*, AD vs. Control group. **(C)** Percentages of ACE2^+^ non-vascular cells among human brain regions of control and AD. *p < *0.05*, AD vs. Control group, Student’s *t*-test.

Furtherly, we compared the expression of ACE2 between healthy control and AD patients in all 12 brain regions. Based on the MOD analysis, ACE2 was downregulated in the basal nucleus, hippocampus, entorhinal cortex, middle frontal gyrus, visual cortex, and amygdala in AD patients. However, no significant difference in MOD was detected in other regions (Figure 3A). To eliminate the signal of endothelial cells, the number of positive non-vascular cells, mostly neurons, according to previous studies (Doobay et al., 2007; Song et al., 2021), was counted in each region. The numbers of ACE2-positive cells except endothelial cells were significantly decreased in the basal nucleus, middle frontal gyrus, and visual cortex in the AD-pathology subjects (Figure 3B). To lower the impact of different cellular number in each section, the percentages of ACE2^+^ cell (the percentage of the number of ACE2^+^ non-vascular cells with the number of non-vascular cell nucleus) within each brain region were further summarized, indicating reduced expression of ACE2 in basal nucleus, hippocampus and entorhinal cortex, middle frontal gyrus, and visual cortex under AD pathology (Figure 3C).

**FIGURE 4:**
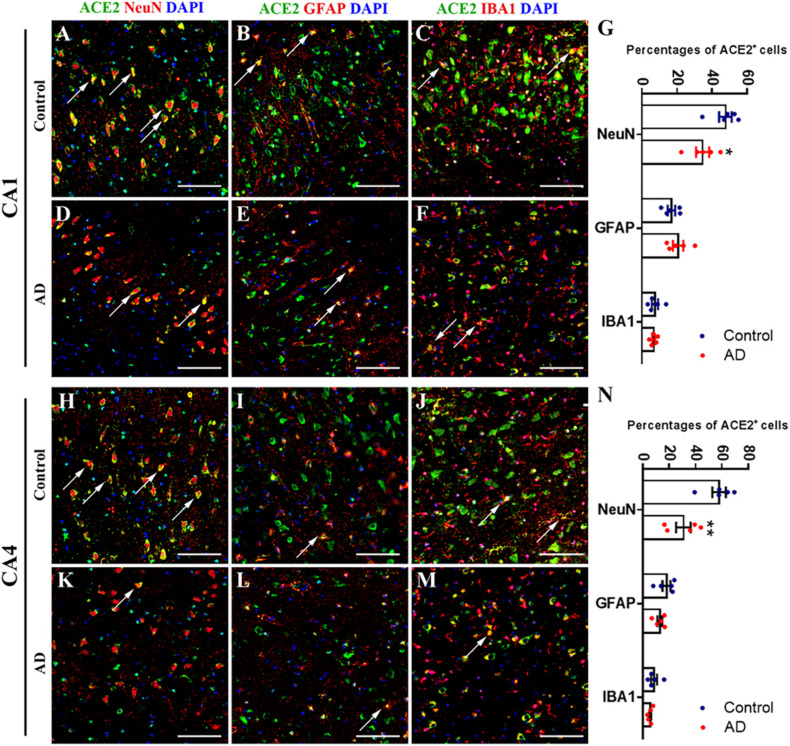
Downregulated neural ACE2 in hippocampus CA1 and CA4 regions of AD subjects. **(A–F)** Immunofluorescence staining of ACE2 with neuronal marker NeuN, astrocytic marker GFAP, and microglial marker IBA1 within CA1 region of control and AD subjects. Scale bar = 100 μm. **(G)** Percentage s of ACE2^+^ cells among NeuN, GFAP, and IBA1 marked cells within CA1 region of control and AD subjects. *p < *0.05*, AD vs. Control group, Student’s *t*-test. **(H–M)** Immunofluorescence staining of ACE2 with neuronal marker NeuN, astrocytic marker GFAP, and microglial marker IBA1 within CA4 region of control and AD subjects. Scale bar = 100 μm. **(N)** Percentages of ACE2^+^ cells among NeuN, GFAP, and IBA1 marked cells within CA4 region of control and AD subjects. **p < *0.01*, AD vs. Control group, Student’s *t*-test.

In order to uncover the cellular type of ACE2^+^ cell, we applied double immunofluorescent labeling in CA1 and CA4 regions. For CA1 region, we detected ACE2 signal in neuron (marked by NeuN), astrocyte (marked by GFAP), and microglia (marked by IBA1), and ACE2 mostly located in neuron (Figures 4A–F). In addition, the percentage of ACE2^+^ neuron in CA1 region was reduced in AD, while no significant difference was detected in astrocyte and microglia (Figure 4G). For CA4 region, ACE2 was also downregulated in neuron without detectable difference in astrocyte and microglia (Figures 4H–N).

## Discussion

COVID-19, the worldwide infectious disease, has been attacking multiple areas in the world with innumerable infections and deaths. In this study, we first mapped the distribution of ACE2 in different brain regions and then uncovered the altered expression of ACE2 in the AD-pathology brain, which provided fundamental knowledge to understand and manage symptoms of COVID-19 patients in the central nervous system.

A series of clinical studies have reported the neurological symptoms in COVID-19 patients, suggesting the invasion of SARS-CoV-2 into the central nervous system (Mao et al., 2020). ACE2, the host cell receptor of SARS-CoV and SARS-CoV-2, was detected in the murine brain and mainly located in the neuronal cell. The expression level of ACE2 varied among mice’s brain regions (Doobay et al., 2007). Also, previous studies found that ACE2 was detected in the human brain and located in endothelial cells (Hamming et al., 2004). However, recent studies detected SARS-CoV-2 in the brain neurons of dead COVID-19 patients and detected the expression of ACE2 in the neuronal cell of organoids, suggesting the expression of ACE2 in brain neuronal cells (Song et al., 2021). In this study, we systematically revealed the expression of ACE2 in the human brain region. All the 12 selected brain regions found the expression of ACE2, suggesting that these regions might all invaded by SARS-CoV-2. The endothelial ACE2 might mediate the virus’s entry to the central nervous system, and the neuronal ACE2 might contribute to the damaged neurological function. In addition, we found that pons, visual cortex, and amygdala expressed a high level of ACE2, suggesting the potential damages in these regions of infected cases. Clinical symptoms, such as mental disturbance and memory deterioration, also echoed the injury in the amygdala (Alonso-Lana et al., 2020). The relative functions of the pons and visual cortex, such as motor regulation and visual formation, should also be taken into consideration in subsequent clinical practice. Previous study also reported that ACE2 was relatively highly expressed in choroid plexus and paraventricular nuclei of the thalamus based on publicly available brain transcriptome databases. These regions were not included in this study, which could be further investigated. It also mentioned that only a few ACE2-expressing nuclei were found in a hippocampal dataset, and none were detected in the prefrontal cortex (Chen R. et al., 2021). The inconsistent results from single nucleus RNA-Seq and immunohistochemistry could be addressed by uncovering the subcellular distribution of ACE2 mRNA and ACE2 protein.

Recent studies also revealed the association between AD and COVID-19. Apart from old age and comorbidities (e.g., hypertension and diabetes), people with AD suffered an increased risk of severe COVID-19 and mortality (Kuo et al., 2020; Zhou et al., 2020; Chen Y. et al., 2021). In addition, AD evoked intensive changes in the protein profile of multiple brain regions, suggesting different scopes of neuronal damage in the central nervous system for AD patients (Xiong et al., 2019). ACE2 plays the central role in the neural infection of SARS-CoV-2, while the expression of ACE2 in the brain with AD-pathology is still controversial. The human brain microarray dataset and Western blot analysis revealed that ACE2 was upregulated in the hippocampus (Lim et al., 2020; Ding et al., 2021). However, another study reported that ACE2 activity in the mid-frontal cortex was significantly reduced in AD compared with age-matched controls and correlated inversely with levels of Aβ and phosphorylated tau (p-tau) pathology (Kehoe et al., 2016). In addition, ACE2 and its byproduct Ang (1–7) were already regarded to be involved in AD pathophysiology. In brief, there are two proposed mechanisms of interaction between AD and ACE2/Ang (1–7) axis of RAS: (1) Ang (1–7) may be biologically active in the brain tissue, where it could exert a neuroprotective role. AD patients may have lower Ang (1–7) levels in key brain regions, contributing to neurodegeneration. (2) Systemic Ang (1–7) is important to cerebral blood flow regulation and blood–brain barrier integrity. Plasma Ang (1–7) seems to be reduced in AD patients (Jiang et al., 2016; Ribeiro et al., 2021). This lack of circulating Ang (1–7) could result in an impaired neurovascular coupling and a disrupted blood–brain barrier (Kehoe, 2018; Wright and Harding, 2019; Ribeiro et al., 2020). In this study, we systemically analyzed the altered expression of ACE2 in AD-associated 12 brain regions. We observed that the ACE2 expression was decreased in the basal nucleus, hippocampus, entorhinal cortex, middle frontal gyrus, visual cortex, and amygdala in brain tissue with AD pathology, suggesting the attenuated function of ACE2, and disturbed the RAS in these regions. As AD high-related brain regions, CA1 and CA4 were selected to identify the cellular type of ACE2^+^ cells. The results indicated that ACE2 mainly expressed in neuron, while also detectable in astrocyte and microglia. Neural ACE2 was downregulated under AD pathology, suggesting the disturbance of RAS system and damaged neural function in these brain regions. Moreover, recent studies revealed that SARS-CoV-2 downregulated the expression of ACE2 in the host cell, and the disturbing ratio of ACE1/ACE2 might be crucial in the pathophysiological process of COVID-19 (Pagliaro and Penna, 2020; Sriram and Insel, 2020; Bank et al., 2021; Beyerstedt et al., 2021). Collectively, we speculated that downregulation of ACE2 might be the shared pathogenesis for both AD and COVID-19, and therapeutic schedules targeting ACE2 might benefit those two special populations.

There might be a mutualistic relationship between AD and COVID-19. On the one hand, the existence of AD-pathology might aggravate the consequence of SARS-CoV-2 infection through the activation of the inflammatory cascade, oxidative stress, possession of APOE ε4, and decreased ACE2/Ang (1–7) protection in the brain. On the other hand, SARS-CoV-2 infection might lead to a higher risk of AD in the future through inducing neuroinflammation, overactivation of the classical RAS, blood–brain barrier damage, ischemic within white matter, and the downregulation of ACE2 (Miners et al., 2020).

## Limitations

This study contained the following limitations. First, the specific mechanism of reduced ACE2 expression in AD pathology was still not identified. Second, we only applied immunohistochemistry to evaluate the level of ACE2, and the application of other technology, such as Western blot and quantitative-PCR, could further sustain our conclusions. Finally, we only selected the female subjects to exclude the interference of gender due to the limited number of appropriate cases, and the clinical record was not detailed. These factors might affect the results, and further preclinical and clinical observations are necessary to uncover the association between ACE2 among brain regions and AD pathophysiology.

## Data Availability Statement

The original contributions presented in the study are included in the article/Supplementary Material, further inquiries can be directed to the corresponding authors.

## Ethics Statement

The studies involving human participants were reviewed and approved by the Institutional Review Board of the Institute of Basic Medical Sciences of the Chinese Academy of Medical Sciences, PUMC, Beijing, China (approval number: 009-2014). The patients/participants provided their written informed consent to participate in this study.

## Author Contributions

HC, SS, and YC drafted the manuscript, performed the immunohistochemistry, and analyzed the data profile. CM and WQ designed this project and revised the manuscript. All authors contributed to the article and approved the submitted version.

## Conflict of Interest

The authors declare that the research was conducted in the absence of any commercial or financial relationships that could be construed as a potential conflict of interest.
